# Supplementary material for: Keystrokes: A practical exploration of semantic drift in timed word association tasks
Source: PLoS One. 2024 Jul 1;19(7):e0305568. doi: 10.1371/journal.pone.0305568 (PMC11216599; doi:10.1371/journal.pone.0305568)
Supplement: S1 Appendix — (DOCX) [file pone.0305568.s001.docx]

# Appendix: Survey

# A Matter of Words

# Short description

# A short, timed, word association study to understand what words mean to everyone, and what they mean to each of us as individuals.

**Free Word Associations: How to answer the questions, and your participation**

# The survey contains six-word associations, and should take no longer than 3 minutes to complete, with reading time included.

# The survey begins with word associations for several CUE words. Basically, traits or qualities you associate with the CUE provided. Try to use single words where possible, though compound adjectives (e.g., sugar-free) are also acceptable. Please do not write full sentences as these will invalidate the submission.

# To avoid overthinking answers, the survey will progress automatically to the next CUE word or question when the timer expires (25 seconds for each CUE).

# Please use a COMMA to separate each unique association, for example, if presented with the word BIRD you might write: *Flying, feathers, ducks*

# Participation

# If you have any ethical concerns about the survey and would like to speak to someone who is not involved please contact the Leeds Business School research ethics liaison and coordinator, Dr Dong Hoang at d.hoang@leedsbeckett.ac.uk.

# We do not collect any personal data in the survey, but we do receive basic demographic data from Prolific as part of their service, and against your Prolific ID. Those IDs will be deleted once processing has completed. The anonymous responses will be made generally available to the research community when the study is published. By participating in this study, which is completely voluntary, you agree to your data being used for the purposes of the study.

**Questions**

Q1 tree: Write as many associations as you can. Separate each with a COMMA.

Q2 love: Write as many associations as you can. Separate each with a COMMA.

Q3 plastic: Write as many associations as you can. Separate each with a COMMA.

Q4 quality: Write as many associations as you can. Separate each with a COMMA.

Q5 dog: Write as many associations as you can. Separate each with a COMMA.
