# Supplementary material for: Keystrokes: A practical exploration of semantic drift in timed word association tasks
Source: PLoS One. 2024 Jul 1;19(7):e0305568. doi: 10.1371/journal.pone.0305568 (PMC11216599; doi:10.1371/journal.pone.0305568)
Supplement: Attachment — Submitted filename: Response to Reviewers.docx [file pone.0305568.s002.docx]

**Response to Reviewers**

| **Reviewer 1** | **Team Response** |
| --- | --- |
| 1. Firstly, the writing style is notably redundant, with numerous instances where the same points are reiterated unnecessarily. This redundancy detracts from the overall readability and coherence of the paper, making it challenging for readers to grasp the core contributions and findings effectively. | We have attempted to address this with several deletions and simplifications. We have reduced our figure count to three, as most of the figures provided did not add new information to the tables that were already provided. Should any more examples have been missed, please don’t hesitate to cite a couple of examples and we will attempt to refine further. |
| 2. A glaring omission in the manuscript is the lack of a comprehensive Related Work section. | This has been amended now with a Related Work Section. This includes a reorganisation of the original intro and new paragraphs. |
| 3 and 4. The exclusive reliance on GloVe embeddings for the analysis is another limitation…Furthermore, the paper could enhance its analysis by incorporating lexical resources like WordNet. | Have now added BERT and WordNet analyses, and updated the methods, results, and discussions accordingly. |
| 5. Lastly, the study's scope, limited to just five cue words, raises questions about its statistical sufficiency and the ability to generalize its findings. A broader selection of words would likely provide a richer and more diverse dataset, potentially yielding more robust and comprehensive insights into semantic drift and word association dynamics. | We have added a limitations section that highlights this. We have also made several references in the discussion to following this research up with a larger set of cues and responses. |
| **Reviewer 2** |  |
| 1. I therefore recommend providing a very simple explanation of the task that participants carried out early on and explicitly mentioning what the quartiles are. | Added to the abstract. |
| 2. It is still not completely clear to me how the semantic drift investigated here relates to other forms of semantic drift. A more explicit explanation would strengthen the paper. | As noted for Reviewer 1, this has been amended now with a Related Word Section This includes a reorganisation of the original intro and new paragraphs. |
| 3. Typos | Corrected |
