# Supplementary material for: Keystrokes: A practical exploration of semantic drift in timed word association tasks
Source: PLoS One. 2024 Jul 1;19(7):e0305568. doi: 10.1371/journal.pone.0305568 (PMC11216599; doi:10.1371/journal.pone.0305568)
Supplement: Attachment — Submitted filename: Response to Reviewers 0530.docx [file pone.0305568.s003.docx]

**Response to Reviewers**

| **Reviewer 2** | **Team Response** |
| --- | --- |
| L411: `the moderate effect sizes we obtained for all pre-registered hypotheses (except H4)’ How about Hypothesis 2? Here, the null hypothesis could not be rejected. I suspect H2 was included since there were effects, but not strong enough to reject the null hypothesis, but as a reader this is confusing. I recommend taking up H2 in the `except’ together with H4, possibly making the difference between the two explicit. | Line 418: Changed to “Although we were unable to reject H2 (stronger effect sizes for abstract versus concrete terms), the moderate and significant effect sizes we obtained for H1, H3 and H5a and b, may be regarded as promising.” |
|  |  |
| I recommend adding a short paragraph to the introduction summing up the main purpose and contribution of the paper. Redundancy between an abstract and introduction are okay. | Line 71: Added a new paragraph “The present study aims to explore this phenomenon in the context of short, free-word-associations tasks. In this study, we seek to explore to what extent meaning might evolve as we engage, cognitively, with a cue (or idea), and how might the process of imagining and remembering, change how far we veer away from common, established associations to personally relevant and meaningful simulations? Importantly, can such shifts in meaning be detected in a sub-minute word association task? And if so, how might that inform the design and analysis of future studies that include or even revolve around such free-word-associations?” |
